# Effect of Aquatic Proprioception Training, Strengthening, and Balance Exercises on Knee Joint Stability and Functional Performance in Anterior Cruciate Ligament Injury

**DOI:** 10.7759/cureus.109064

**Published:** 2026-05-17

**Authors:** Manaswi M Shaha, Sandeep Shinde, Akshaya V Joshi, Manoj Ambali

**Affiliations:** 1 Department of Musculoskeletal Sciences, Krishna College of Physiotherapy, Krishna Vishwa Vidyapeeth (Deemed to be University), Karad, IND; 2 Department of Anatomy, Krishna Institute of Medical Sciences, Krishna Vishwa Vidyapeeth (Deemed to be University), Karad, IND

**Keywords:** anterior cruciate ligament injury, aquatic therapy, functional performance, knee joint stability, proprioception

## Abstract

Background

Anterior cruciate ligament (ACL) injuries frequently induce symptomatic knee instability and functional decline among active individuals. This pathology extends beyond mechanical insufficiency, involving significant deficits in muscle force production, balance, and proprioception. Collectively, these neuromuscular disruptions impair joint stability and represent critical factors that can delay the recovery of full functional status. While conventional land-based rehabilitation remains the standard approach, aquatic rehabilitation has gained attention due to its ability to reduce joint loading while enhancing neuromuscular control through the unique physical properties of water. However, evidence evaluating the combined effect of aquatic proprioceptive training, strengthening, and balance exercises on functional outcomes following ACL injury remains limited.

Objectives

The primary objective of this study is to evaluate the impact of a structured six-week aquatic-based rehabilitation protocol, which integrates proprioceptive, strengthening, and balance exercises, on knee joint stability and functional performance in patients diagnosed with grade 1 and 2 ACL injuries. Furthermore, the research involves a comparative analysis to determine the efficacy of this water-based intervention relative to a traditional land-based rehabilitation strategy.

Methods

This experimental study analysed 120 patients, aged 18 to 35, with grade 1 and 2 ACL tears who received non-operative treatment rather than surgery. Participants were randomly allocated into two groups: Group A (aquatic rehabilitation) and Group B (land-based rehabilitation), with 60 participants in each group. Participants in both study arms engaged in a systematic rehabilitation protocol, attending three sessions weekly over a total duration of six weeks. The study evaluated four primary parameters: proprioception (joint position sense), strength (one-repetition maximum (1RM) leg press), dynamic balance (Y Balance Test), and self-reported function assessed using the International Knee Documentation Committee (IKDC) score. Changes from baseline to post-intervention were scrutinised using paired t-tests, while differences between groups were assessed via independent t-tests. All statistical analyses utilised a stringent significance level of p <0.0001.

Results

Although both rehabilitation modalities significantly improved knee function and neuromuscular control (p <0.0001), the aquatic program demonstrated clear superiority. Participants in the aquatic group achieved significantly greater improvements in proprioception, muscle strength, and dynamic balance compared to the land-based control. The resulting higher IKDC scores underscore the aquatic environment's enhanced efficacy in restoring knee stability and overall functional capacity in patients with ACL injuries.

Conclusion

Aquatic-based rehabilitation, incorporating proprioceptive and resistance training, demonstrates significantly greater improvement than conventional land-based therapy in optimising knee stability and functional outcomes for patients with ACL injuries. The findings support the integration of structured aquatic rehabilitation programs into ACL management protocols to optimise neuromuscular recovery and functional outcomes.

## Introduction

The knee joint represents a complex synovial structure subjected to significant mechanical loading. It is critical for the execution of various weight-bearing movements, ranging from steady-state walking to high-velocity manoeuvres such as jumping and rapid deceleration or change of direction in sporting contexts. The integrity of knee function depends on the coordinated interaction of passive stabilisers, active muscular support, and neuromuscular control mechanisms that collectively maintain joint stability during dynamic movement [1]. Among the passive stabilisers, the anterior cruciate ligament (ACL) is a critical structure responsible for restraining anterior tibial translation and providing rotational stability to the knee, particularly during high-demand activities [2]. Injury to the ACL disrupts these stabilising mechanisms, leading to joint instability, altered movement strategies, and compromised functional performance [3].

Ruptures of the ACL are frequently documented among athletic and youthful cohorts. These injuries are particularly prevalent in sporting environments that necessitate frequent pivoting, sharp cutting manoeuvres, and rapid deceleration, all of which place significant torsional stress on the knee. Epidemiological evidence indicates a rising incidence of ACL injuries globally, with substantial physical, psychological, and economic consequences [1,4]. Beyond the immediate mechanical laxity, ACL injuries are characterised by persistent impairments in proprioceptive feedback, muscular force production, and neuromuscular control. These deficits often endure regardless of whether the patient undergoes surgical reconstruction or follows a non-operative path. Such chronic dysfunction not only compromises dynamic joint stability but also serves as a primary risk factor for secondary injury, significantly hindering the patient’s ability to resume pre-injury athletic or functional levels [5-7].

Management of ACL injury typically includes either surgical reconstruction or non-operative rehabilitation, with the choice of treatment modulated by the intensity and frequency of the individual's physical exertion, functional instability, and patient goals [8]. Longitudinal data suggest that therapeutic equivalence in functional recovery is attainable across both management modalities when they are supported by the implementation of a well-organised physical therapy program [9]. Regardless of the management strategy, rehabilitation plays a decisive role in restoring knee joint stability and functional performance. Consequently, contemporary ACL rehabilitation emphasises neuromuscular recovery rather than focusing solely on structural healing [10-14].

Proprioception, which may be defined as the afferent input arising from mechanoreceptors within muscles, ligaments, and joint capsules, is essential for joint position sense and movement control [7]. Following ACL injury, damage to ligamentous mechanoreceptors results in impaired proprioceptive feedback, which adversely affects sensorimotor integration and dynamic knee stability [8]. Consequently, proprioceptive interventions have emerged as a fundamental component of ACL recovery protocols. These exercises are designed to refine kinesthetic awareness, bolster neuromuscular regulation, and facilitate the adoption of safer, more efficient biomechanical strategies during weight-bearing activities. Systematic reviews and meta-analyses have reported that proprioceptive interventions significantly improve knee function and postural control in individuals following ACL reconstruction [15-17].

Muscle strengthening, particularly of the quadriceps and hamstrings, is another fundamental component of ACL rehabilitation, as these muscle groups contribute to dynamic stabilisation of the knee joint [10]. Deficits in muscle strength following ACL injury or reconstruction have persisted, associated with reduced functional performance and increased risk of reinjury. Balance training further complements rehabilitation by improving postural stability, coordination, and dynamic control, which are essential for safe return to activity [18]. The integration of proprioceptive, strengthening, and balance exercises has been shown to produce superior functional outcomes compared to isolated interventions [19].

Throughout the last 10 years, there has been an intensifying research focus on the clinical utility of aquatic rehabilitation for ACL-deficient populations. Current evidence suggests that this approach serves as a viable and effective alternative or adjunct to traditional terrestrial physical therapy. The unique physical properties of water, including buoyancy, viscosity, and hydrostatic pressure, allow for reduced joint loading while simultaneously providing resistance and enhanced sensory feedback [20]. These characteristics enable early initiation of functional movements with reduced pain and fear of movement, while promoting neuromuscular activation and postural control. Aquatic environments also offer a safe and controlled setting for balance and proprioceptive training, particularly during the early and intermediate phases of rehabilitation [14].

Emerging evidence suggests that aquatic-based rehabilitation programs can produce outcomes comparable to, or in some cases superior to, traditional land-based rehabilitation in terms of knee stability, muscle strength, and functional performance [21-23]. However, despite growing interest in aquatic therapy, there remains limited research examining the combined effects of aquatic proprioceptive training, strengthening, and balance exercises as an integrated rehabilitation approach for ACL injury. Furthermore, variability in rehabilitation protocols and outcome measures highlights the need for structured research focusing on comprehensive aquatic rehabilitation strategies [16].

Reliable and valid outcome measures are essential for evaluating rehabilitation effectiveness. Tools such as joint position sense (JPS) testing for proprioception, one-repetition maximum (1RM) leg press testing for muscle strength, the Y Balance Test for dynamic balance, and the International Knee Documentation Committee Score (IKDC) score for functional performance have demonstrated strong psychometric properties in individuals with knee disorders, including ACL injury [9-12]. The use of these outcome measures allows for objective and subjective assessment of rehabilitation outcomes and supports evidence-based clinical decision-making.

Given the increasing incidence of ACL injuries and the growing emphasis on neuromuscular rehabilitation, there is a clear need to investigate innovative rehabilitation approaches [24-26]. Investigating the efficacy of aquatic-based proprioceptive, strengthening, and balance interventions is essential for refining evidence-based rehabilitation strategies for ACL-deficient populations. Consequently, this study was designed to quantify the impact of an integrated aquatic exercise protocol on knee stability and functional outcomes in patients recovering from ACL trauma. We hypothesised that patients participating in an integrated aquatic rehabilitation program would demonstrate significantly greater improvements in proprioception (JPS), lower limb strength (1RM leg press), dynamic balance (Y Balance Test), and functional performance (IKDC) compared to those following a land-based protocol.

## Materials and methods

This was a comparative study conducted at Krishna Vishwa Vidyapeeth (Deemed to be University), Karad. Samples were calculated using the formula $n = \frac{Z^{2}pq}{L^{2}}$, where n represented the sample size; the Z value was set to 1.96, which corresponds to a 95% confidence interval; p represented the estimated prevalence of ACL injury is 55% [4]; q was calculated as 100-p, resulting in 45%; and L denoted the allowable error is 8.63%, the expected sample size estimated using the formula was 128 participants.

A total of 128 participants from the local region, aged 18-35 years and clinically diagnosed with grade 1 and 2 ACL injury by an orthopaedic surgeon, were recruited for the study. Conversely, individuals were excluded if they had concomitant fractures, multi-ligament trauma, or recent lower-limb surgery (excluding prior ACL reconstruction). Other exclusion criteria included acute febrile illnesses, aquaphobia, and dermatological conditions such as open wounds or skin infections.

A total of 128 individuals were initially evaluated for eligibility to meet the expected sample size requirement. Of these, eight individuals were excluded from the final study: five participants did not meet the specific inclusion parameters, and three participants declined to provide consent. This resulted in a final sample size of 120 participants. Notably, there were no dropouts reported during the six-week intervention period following the initial allocation.

Participants were divided into two intervention groups using a simple randomisation technique. Allocation was done using the envelope method, where two envelopes labelled as Group A and Group B were prepared. Before the start of the intervention, each participant was instructed to pick one envelope, which determined their group assignment. This method ensured an impartial and unbiased distribution of participants across both groups.

**Figure 1:**
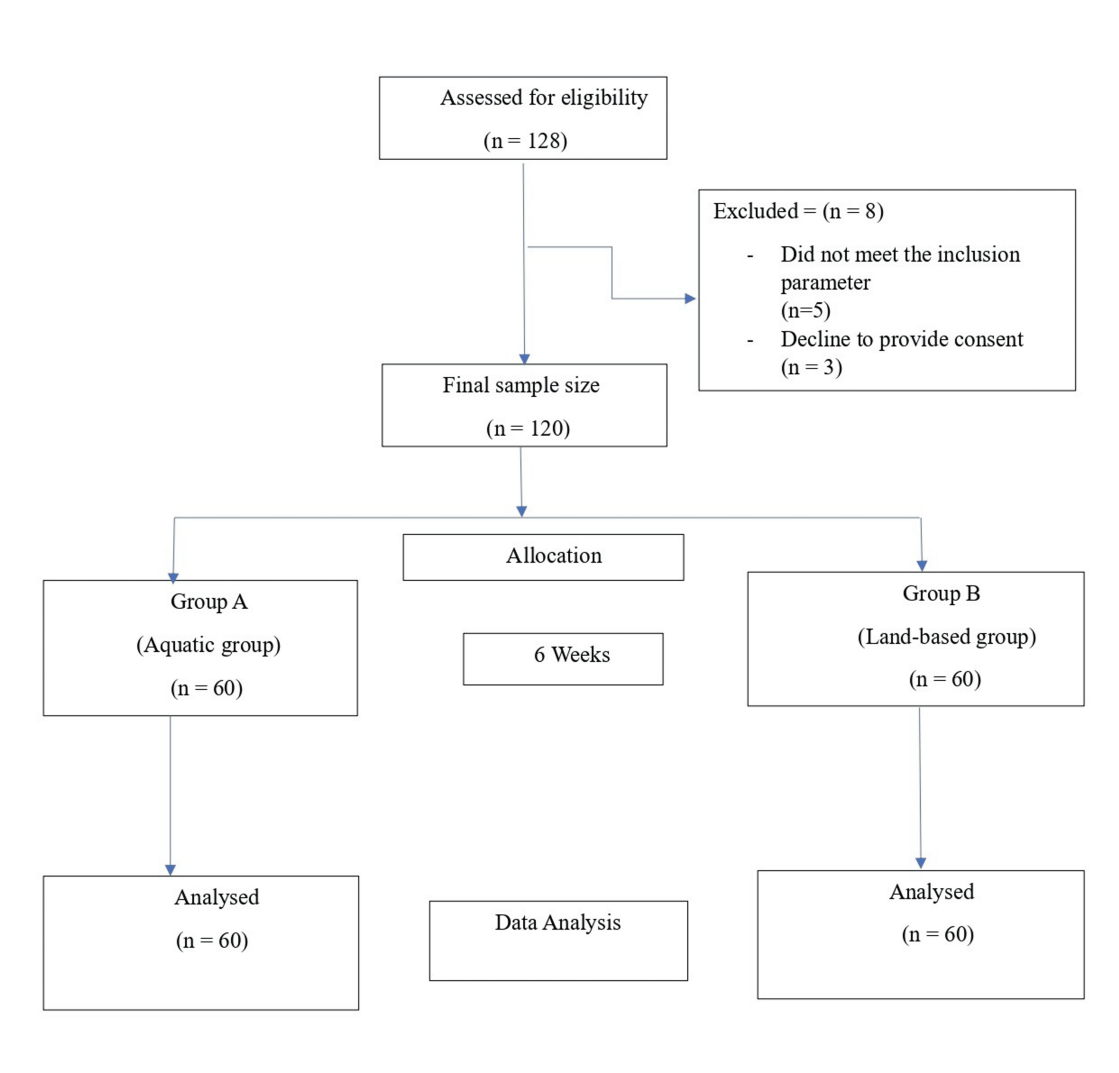
Study flowchart

Because the distinction between aquatic and land-based interventions was obvious, neither the participants nor the assessors could be blinded to group assignments. To minimise potential bias, we employed procedural blinding by withholding specific hypotheses and details of the alternative protocol from the participants. Additionally, we used strict scheduling and a separate location to ensure no contact occurred between groups, thereby preventing treatment contamination. Figure 1 provides a study flowchart.

Before initiating the research, ethical clearance was granted by the Institutional Ethics Committee of Krishna Vishwa Vidyapeeth (Deemed to be University), Karad (protocol number 364/2025-2026). The study was conducted in full compliance with the ethical standards of the Declaration of Helsinki. Prior to participation, all subjects provided informed written consent, having been guaranteed complete data privacy and the freedom to withdraw from the research at any point without any negative consequences.

Outcome measures

JPS Test

Proprioception was assessed using the JPS test, which evaluates the individual’s ability to perceive and reproduce a predetermined knee joint angle. The participant was positioned in a sitting position with the hip and knee supported and the lower limb relaxed. Vision was eliminated using an eye mask to prevent visual feedback. The examiner passively moved the participant’s involved knee to a target angle within the mid-range of knee flexion and held the position for a few seconds to allow the participant to memorise the joint position. The limb was then returned to the starting position. The participant was instructed to actively reproduce the target knee angle as accurately as possible. The difference between the target angle and the reproduced angle was recorded in degrees using a goniometer. Three trials were performed, and the mean absolute error was determined for analysis. Lower error values indicated better proprioceptive accuracy [10].

One-Repetition Maximum (1RM) Test

Lower body strength was assessed using a submaximal repetition-to-failure protocol to estimate the 1RM on a leg press. An initial weight closely approximating the participant's estimated 1RM was selected. The participant was then instructed to perform as many continuous leg press repetitions as possible until muscular failure. If the participant successfully completed more than 12 repetitions, a 15-minute rest period was provided, the weight was increased, and the testing protocol was repeated to ensure accuracy [11].

Y Balance Test

The Y Balance Test was employed as a metric for single-limb equilibrium and sensorimotor coordination. Standing on the involved leg, subjects executed maximal reaches in three standardised directions (anterior, posteromedial, and posterolateral). To ensure data reproducibility, three attempts were recorded per direction, and the test was repeated to verify consistency. Raw reach distances were then adjusted relative to anatomical limb length to eliminate height-related bias. This normalisation process allowed for a standardised comparison of dynamic balance, where increased reach excursion reflected enhanced neuromuscular performance [12].

IKDC Score

The IKDC subjective form was utilised as a primary passive range of motion to evaluate the impact of knee disorders on physical performance and symptomatic expression. Participants provided self-reported data regarding their functional status during the preceding period, focusing on sports-related and routine daily tasks. Following the standardised IKDC guidelines, responses were aggregated into a final score between 0 and 100. This scoring system is interpreted such that an increase in the total score represents an amelioration of symptoms and improved functional integrity [13].

The treatment program consisted of three phases: warm-up, active exercise training, and cool-down. A six-week intervention window was utilised for both cohorts, with exercise sessions administered at a tri-weekly frequency to ensure consistent physiological loading. Exercise progression was implemented from week 1 to week 6 based on patient tolerance and performance.

**Figure 2:**
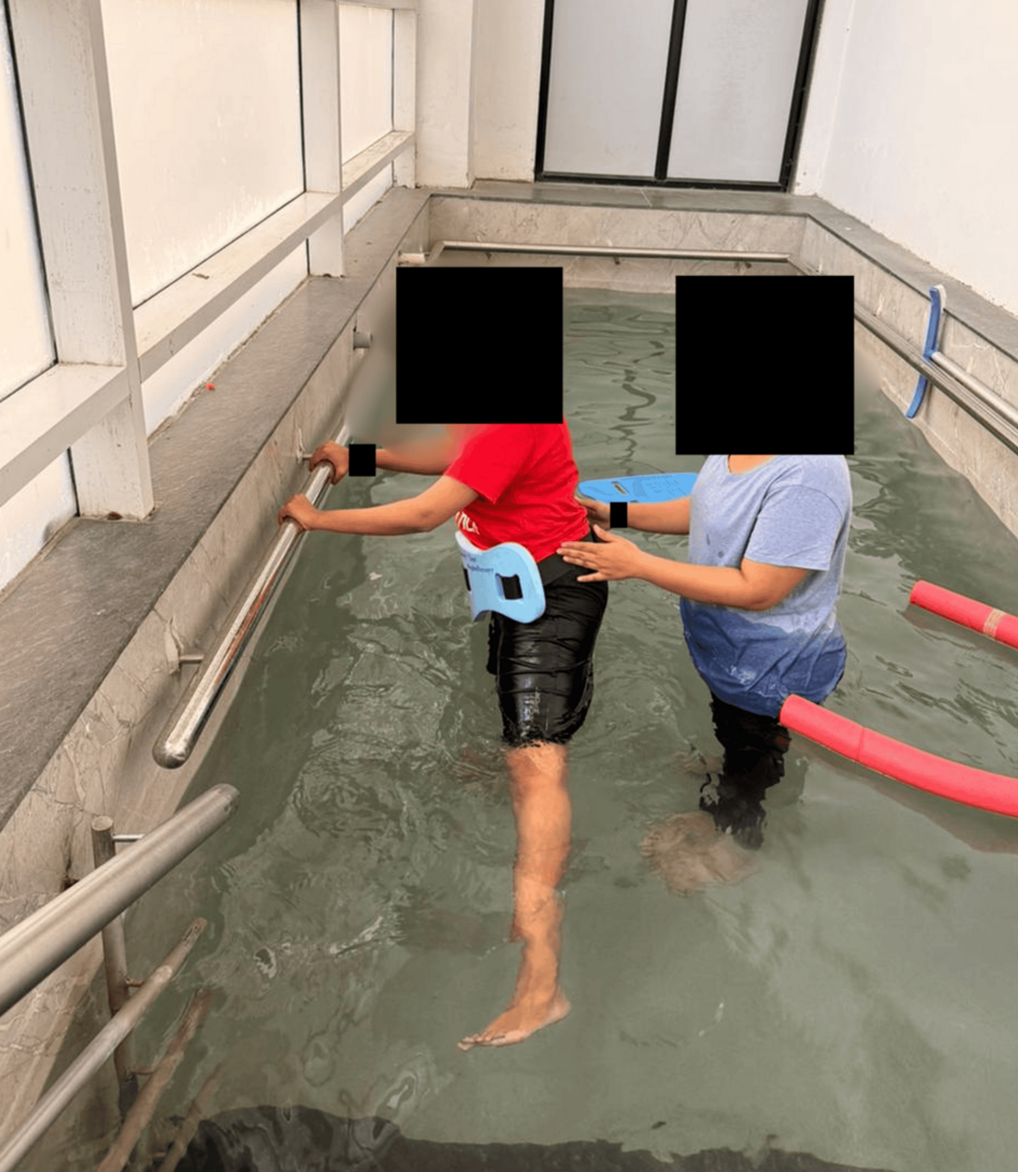
Patient performing aquatic exercise

**Figure 3:**
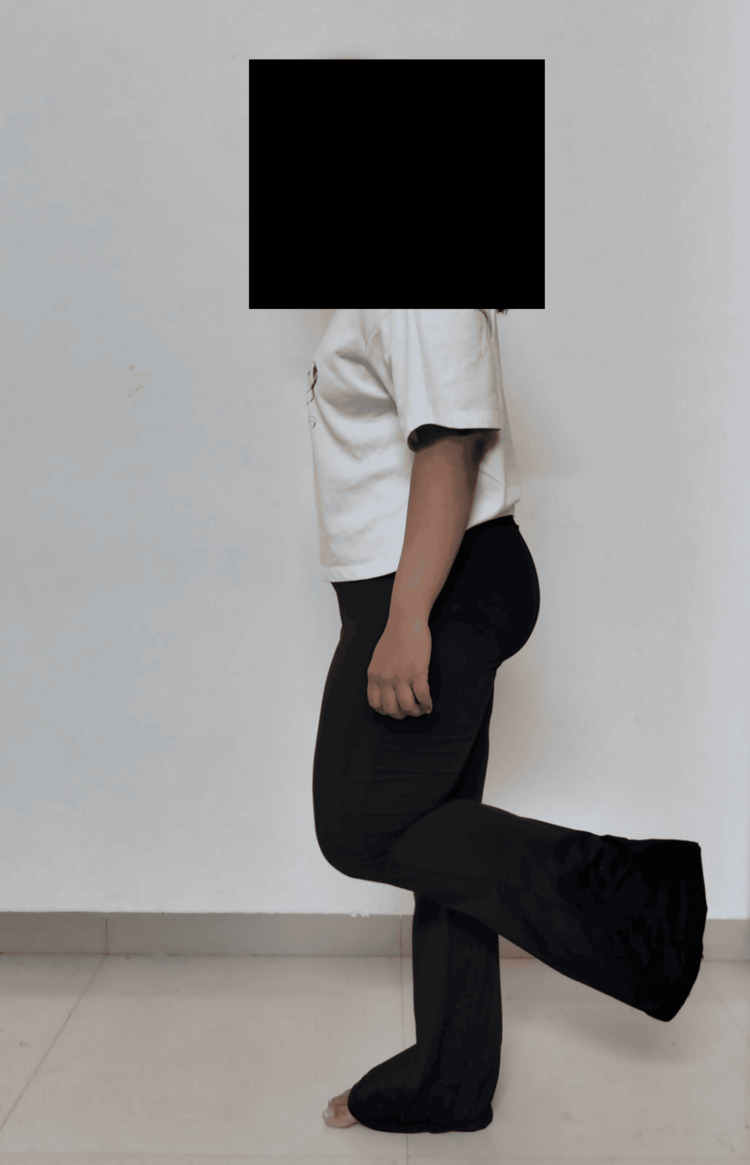
Patient performing land-based exercise

The water temperature was maintained between 32°C and 36°C, with immersion up to mid-sternum level. Hydrotherapy equipment, such as aquatic weight cuffs and noodles, was utilised. All sessions were conducted under the supervision of a licensed physiotherapist, with safety as a priority. Exercise intensity was continuously adjusted based on participant tolerance and clinical judgment. Table 1 outlines the six-week treatment protocols for both Group A (aquatic rehabilitation) and Group B (land-based rehabilitation). The implementation of the aquatic and land-based exercise protocols is demonstrated in Figures 2-3, respectively.

**Table 1:** Treatment protocol for Groups A (aquatic rehabilitation) and B (land-based rehabilitation) from one to six weeks

Type of Exercise	Aquatic Exercise	Repetitions/Sets (Weeks 1-6)	Land-Based Exercise	Repetitions/Sets
Warm-up exercise (10 min)	Active range of motion of lower limb; Walking; Marching; Heel raise; Toe raise	10 repetitions × 3 sets	Active range of motion of lower limb; walking; marching; heel raise; toe raise	10 repetitions × 3 sets
Proprioception exercise	Wall press; mini squats 20-30 degree; step-up; step-down; forward and backward lunges; hopping; cone walking	10 repetitions × 3 sets	Wall press; mini squats 20-30 degree; step-up; step-down; forward and backward lunges; hopping; cone walking	10 repetitions × 3 sets
Strengthening exercise	Single leg raise; mini squats with ball press; hip abduction adduction with weights; hamstring concentric and eccentric contraction; cycling; side stepping; sit to stand; resisted band walking	10 repetitions × 3 sets	Single leg raise; mini squats with ball press; hip abduction adduction with weights; hamstring concentric and eccentric contraction; cycling; side stepping; sit to stand; resisted band walking	10 repetitions × 3 sets
Balance exercise	Single leg stance; tandem walking; Y-balance exercises; diagonal reach exercise; box jump; Bosu balance training; weight shifts; trunk rotation	10 repetitions × 3 sets	Single leg stance; tandem walking; Y-balance exercises; diagonal reach exercise; box jump; Bosu balance training; weight shifts; trunk rotation	10 repetitions × 3 sets
Cool down exercise	Stretching of hamstring; stretching of quadriceps; stretching of adductors; stretching of abductors; stretching of gastrocnemius	4-5 minutes, 10-second hold × 3 sets	Stretching of hamstring; stretching of quadriceps; stretching of adductors; stretching of abductors; stretching of gastrocnemius	4-5 minutes, 10-second hold × 3 sets

Statistical analysis

Data were processed using IBM SPSS Statistics for Windows, Version 26 (Released 2018; IBM Corp., Armonk, New York, United States) and validated through manual analysis. We presented numerical outcomes as means and standard deviations, utilising paired t-tests for within-group pre- and post-intervention comparisons. To ensure the reproducibility of clinical outcomes and minimise type I error inflation, a conservative alpha level of p < 0.0001 was established as the criterion for statistical significance.

## Results

A total of 120 individuals with conservatively managed ACL injury participated in the study. Participants were equally divided into two groups, with 60 individuals assigned to the aquatic rehabilitation group (Group A) and 60 to the land-based rehabilitation group (Group B).

Table 2 summarises the baseline demographic and clinical characteristics of the participants. Both groups were well-matched at the start of the study. Most participants in each group were between 26 and 35 years of age. Gender distribution was equal, with males and females representing an identical proportion in both groups. The side of knee involvement was evenly distributed between right and left knees, and a similar proportion of participants in each group were assessed within six weeks of injury. These findings indicate that the two groups were comparable prior to the intervention.

**Table 2:** Demographic and clinical characteristics of the study participants

Variable	Group A - Aquatic (%)	Group B - Land-Based (%)
Age (years)		
18-25	26.7	26.7
26-35	66.7	66.7
36-45	6.6	6.6
Gender		
Male	50.0	50.0
Female	50.0	50.0
Side involved		
Right knee	50.0	50.0
Left knee	50.0	50.0
Duration of injury		
≤6 weeks	51.7	51.7
>6 weeks	48.3	48.3

The results of the JPS test indicate that both groups achieved a statistically significant reduction. However, Group A showed a more substantial clinical improvement, with a mean error reduction of 2.230 compared to 1.690 in Group B. The narrow standard deviations suggest that the treatment response was highly uniform across the participants, confirming that the intervention for Group A is the more effective protocol for enhancing joint proprioception. Table 3 illustrates the comparative pre- and post-intervention mean scores for the JPS test in both Group A and Group B.

**Table 3:** Comparison of pre- and post-test mean scores of joint position sense test within Groups A and B A paired t-test was used to calculate within-group analysis and p-values. SD: standard deviation

Groups	Pre-test (Mean ± SD)	Post-test (Mean ± SD)	Mean Difference	P-value	T value
Group A	4.56 ± 0.04	2.33 ± 0.04	2.230	<0.0001	186.52
Group B	4.43 ± 0.09	2.74 ± 0.09	1.690	<0.0001	297.81

The 1-RM Leg Press results demonstrate that both Group A and Group B experienced statistically significant improvements in lower extremity strength. At the conclusion of the study, Group A exhibited a superior increase in force-generating capacity, with a mean improvement of 21.115 units compared to 17.287 units in Group B. Table 4 illustrates the comparative pre- and post-intervention mean scores for the 1 RM Leg Press Test in both Group A and Group B.

**Table 4:** Comparison of pre- and post-test mean scores of 1RM Leg Press Test within Groups A and B A paired t-test was used to calculate within-group analysis and p-values. SD: standard deviation; 1RM: one-repetition maximum

Groups	Pre-test (Mean ± SD)	Post-test (Mean ± SD)	Mean Difference	P-value	T value
Group A	34.4 ± 0.14	55.5 ± 0.13	21.115	<0.0001	2480.6
Group B	34.9 ± 0.19	52.25 ± 0.19	17.287	<0.0001	1849.3

In alignment with the results from the 1-RM Leg Press and JPS tests, the Y Balance Test confirms the overall superiority of Group A. By achieving the highest gain in dynamic reach distance, Group A has demonstrated the most comprehensive improvement across proprioception, strength, and balance. Table 5 illustrates the comparative pre- and post-intervention mean scores for the Y Balance Test in both Group A and Group B.

**Table 5:** Comparison of pre- and post-test mean scores of Y Balance Test within Groups A and B A paired t-test was used to calculate within-group analysis and p-values. SD: standard deviation

Groups	Pre-test (Mean ± SD)	Post-test (Mean ± SD)	Mean Difference	P-value	T value
Group A	17.08 ± 0.14	32.28 ± 0.18	15.200	<0.0001	1065.8
Group B	17.45 ± 0.22	29.96 ± 0.22	12.508	<0.0001	1501.0

The results demonstrate a substantial improvement in patient-reported knee function and symptom relief for both cohorts, as evidenced by the IKDC subjective evaluation scores. Both groups began the study at a comparable functional baseline, yet both achieved a statistically significant progression toward better outcomes by the conclusion of the treatment. While the recovery was positive across the board, the data indicate that Group A experienced a more robust and consistent enhancement in their physical status compared to Group B. Table 6 illustrates a comparison of pre- and post-test mean scores of IKDC within Groups A and B.

**Table 6:** Comparison of pre- and post-test mean scores of IKDC within Groups A and B A paired t-test was used to calculate within-group analysis and p-values. SD: standard deviation; IKDC: International Knee Documentation Committee

Groups	Pre-test (Mean ± SD)	Post-test (Mean ± SD)	Mean Difference	P-value	T value
Group A	45.0 ± 0.24	72.46 ± 0.19	27.44	<0.0001	2770.5
Group B	45.9 ± 0.23	71.02 ± 0.24	25.08	<0.0001	578.37

The comparative analysis of post-intervention outcomes reveals that Group A achieved significantly better results across all physiological and functional metrics compared to Group B. Specifically, Group A demonstrated superior performance in the JPS test, 1RM Leg Press Test, and Y Balance Test. These physical gains were mirrored by higher IKDC scores, indicating a more favourable subjective recovery of knee function and symptom management. Table 7 illustrates the comparison of post-test mean scores of the outcome measure between Groups A and B.

**Table 7:** Comparison of post-test mean scores of the outcome measure between Groups A and B JPST: joint position sense test, 1RM: one-repetition maximum, IKDC: International Knee Documentation Committee

Outcome Measures	Group A (Mean ± SD)	Group B (Mean ± SD)	P-value
JPST	2.33 ± 0.04	2.74 ± 0.09	<0.0001
1RM Leg Press Test	55.5 ± 0.13	52.2 ± 0.19	<0.0001
Y Balance Test	32.28 ± 0.18	29.96 ± 0.22	<0.0001
IKDC score	72.46 ± 0.19	71.02 ± 0.24	<0.0001

## Discussion

The present study aimed to evaluate the effectiveness of an aquatic-based rehabilitation program on proprioception, muscle strength, balance, and functional performance in individuals with ACL injury. A total of 120 participants were equally allocated into aquatic and land-based rehabilitation groups. Outcome measures, including JPS, 1RM leg press, Y Balance Test, and IKDC score, were utilised, all of which are well-established, reliable, and valid tools for assessing knee joint function and neuromuscular performance [9-12].

Proprioceptive impairment is a well-recognised consequence of ACL injury and plays a critical role in the development of functional instability. Paterno highlighted that sensorimotor deficits may persist despite rehabilitation and contribute to an increased risk of reinjury if not adequately addressed [5]. Similarly, Relph et al. reported significant deficits in JPS and neuromuscular control following ACL injury [7]. Supporting this, Gokeler et al. and Hajouj et al. emphasised that proprioceptive-focused interventions significantly improve joint stability and sensorimotor function in ACL-deficient individuals [3,17].

In the present study, both groups demonstrated improvement in proprioception; however, the aquatic group showed significantly greater reduction in JPS error. This enhanced recovery can be attributed to the unique properties of water, such as hydrostatic pressure and viscosity, which provide continuous afferent stimulation and facilitate neuromuscular re-education. Participants in the land-based group showed significant improvements in JPS. This recovery is likely driven by ground reaction forces and weight-bearing exercises that stimulate mechanoreceptors within the joint capsule and surrounding ligaments.

Muscle weakness, particularly of the quadriceps, is another major impairment following ACL injury and is closely associated with reduced functional capacity. Furthermore, Buckthorpe et al. emphasised the importance of restoring quadriceps strength to achieve optimal knee stability and return to activity [14]. In the present study, both rehabilitation approaches resulted in significant strength gains; however, the aquatic group exhibited superior improvements. This may be explained by the reduced joint loading due to buoyancy, which allows earlier initiation of strengthening exercises, combined with the uniform resistance provided by water. Strength gains in the land-based group were achieved through traditional resistance training, which utilises gravity and progressive loading. This remains the gold standard for developing eccentric control and bone mineral density, both vital for long-term joint health.

Balance and neuromuscular control are essential components for restoring dynamic knee stability and preventing secondary injuries, as deficits in these areas contribute significantly to functional instability following ACL injury [5]. In the present study, both groups demonstrated significant improvements in Y Balance Test performance, though the aquatic group exhibited superior outcomes. While land-based rehabilitation is vital for mimicking the specific athletic demands of ground-reaction forces and directional cutting, the aquatic environment offers a unique advantage through constant fluid perturbations and multidirectional resistance [16,18]. These water-based properties provide a more holistic challenge to the postural control system, forcing continuous adaptive neuromuscular responses that appear to retrain dynamic stability more efficiently than traditional land-based exercises during the middle phases of recovery.

Functional performance, as measured by the IKDC subjective knee form, showed significant post-intervention improvements across both rehabilitation cohorts. The land-based group achieved substantial gains, demonstrating that traditional protocols effectively restore the patient's perception of knee function and facilitate a return to daily activities through gravity-dependent, functional movement patterns [22]. However, the aquatic group exhibited slightly higher overall scores, suggesting a marginal advantage in patient-reported outcomes.

Based on the findings of this study, it can be concluded that both land-based and aquatic rehabilitation programs are effective in improving knee stability and functional performance. However, aquatic rehabilitation demonstrated superior improvements across proprioception, muscle strength, balance, and functional outcomes. The unique physical and sensory properties of water provide an optimal environment for safe, progressive, and comprehensive neuromuscular rehabilitation.

Strengths

A major strength of this study is its comprehensive evaluation of ACL rehabilitation outcomes, including objective measures of proprioception, strength, and balance, as well as a validated patient-reported functional outcome measure. The structured and progressive rehabilitation protocol applied in both groups enhances the clinical relevance of the findings. Furthermore, the study contributes to the growing body of evidence supporting aquatic rehabilitation as an effective modality in ACL injury management.

Limitations

The study was conducted at a single centre over a relatively short six-week duration, which limits the generalisability of the results and leaves the long-term persistence of improvements. The novelty of the aquatic environment may have introduced a Hawthorne effect, where increased participant motivation rather than the physical properties of the water itself influenced outcomes, a factor further complicated by the inherent inability to blind participants and therapists to the treatment groups. Additionally, the principle of training specificity suggests that improvements gained in a buoyant, fluid environment may not translate seamlessly to the high-impact, ground-reaction demands of land-based athletics. Future research utilising multi-centre designs and longitudinal follow-ups is necessary to determine how integrated aquatic-land protocols affect long-term joint health and physical performance.

Future recommendations

Future studies should include longer intervention periods and extended follow-up assessments to evaluate the long-term effects of aquatic rehabilitation on knee stability and functional outcomes. Research involving diverse populations, varying activity levels, and post-surgical ACL reconstruction patients would further enhance the applicability of these findings. Comparative studies examining the optimal timing and integration of aquatic and land-based rehabilitation components may also help refine evidence-based rehabilitation protocols.

## Conclusions

The present study demonstrates that an aquatic rehabilitation program incorporating proprioceptive training, strengthening, and balance exercises leads to significant improvements in knee joint stability and functional performance in individuals with ACL injury. Although both land-based and aquatic rehabilitation were effective, aquatic rehabilitation resulted in superior gains across proprioception, muscle strength, balance, and functional performance as measured by the IKDC score. The supportive and resistance-based properties of water allow effective neuromuscular training while minimising joint stress, making aquatic therapy a safe and effective rehabilitation option. These findings support the inclusion of aquatic rehabilitation as a valuable complement to conventional land-based programs in the management of ACL injury.
